# Periodontitis‐associated pathogens *P. gingivalis* and *A. actinomycetemcomitans* activate human CD14^+^ monocytes leading to enhanced Th17/IL‐17 responses

**DOI:** 10.1002/eji.201545871

**Published:** 2016-08-01

**Authors:** Wan‐Chien Cheng, Saskia D. van Asten, Lachrissa A. Burns, Hayley G. Evans, Gina J. Walter, Ahmed Hashim, Francis J. Hughes, Leonie S. Taams

**Affiliations:** ^1^ Division of ImmunologyInfection & Inflammatory DiseaseCentre for Molecular and Cellular Biology of InflammationKing's College LondonLondonUK; ^2^ Department of PeriodontologyDental InstituteKing's College LondonLondonUK; ^3^ Department of PeriodontologySchool of DentistryTri‐Service General Hospital and National Defense Medical CenterTaipeiTaiwan; ^4^ Centre for Immunology and Infectious DiseaseBlizard InstituteBarts and The London School of Medicine and DentistryQueen Mary University of LondonLondonUK

**Keywords:** *Aggregatibacter actinomycetemcomitans*, Interleukin‐17, Periodontal disease, *Porphyromonas gingivalis*, T helper 17 cells

## Abstract

The Th17/IL‐17 pathway is implicated in the pathogenesis of periodontitis (PD), however the mechanisms are not fully understood. We investigated the mechanism by which the periodontal pathogens *Porphyromonas gingivalis* (*Pg*) and *Aggregatibacter actinomycetemcomitans* (*Aa*) promote a Th17/IL‐17 response in vitro, and studied IL‐17^+^ CD4^+^ T‐cell frequencies in gingival tissue and peripheral blood from patients with PD versus periodontally healthy controls. Addition of *Pg* or *Aa* to monocyte/CD4^+^ T‐cell co‐cultures promoted a Th17/IL‐17 response in vitro in a dose‐ and time‐dependent manner. *Pg* or *Aa* stimulation of monocytes resulted in increased CD40, CD54 and HLA‐DR expression, and enhanced TNF‐α, IL‐1β, IL‐6 and IL‐23 production. Mechanistically, IL‐17 production in *Pg*‐stimulated co‐cultures was partially dependent on IL‐1β, IL‐23 and TLR2/TLR4 signalling. Increased frequencies of IL‐17^+^ cells were observed in gingival tissue from patients with PD compared to healthy subjects. No differences were observed in IL‐17^+^ CD4^+^ T‐cell frequencies in peripheral blood. In vitro, *Pg* induced significantly higher IL‐17 production in anti‐CD3 mAb‐stimulated monocyte/CD4^+^ T‐cell co‐cultures from patients with PD compared to healthy controls. Our data suggest that periodontal pathogens can activate monocytes, resulting in increased IL‐17 production by human CD4^+^ T cells, a process that appears enhanced in patients with PD.

## Introduction

Periodontitis (PD) is a chronic inflammatory disease of the supporting tissues of the teeth, which results in progressive irreversible damage to the alveolar bone and tooth support and ultimately may result in tooth loss. It is caused by the accumulation of bacterial plaque biofilm on the teeth and the host immune responses that occur in the tissues in response to this.

The adaptive T‐cell immune response results in the production of cytokines that may be protective against periodontal disease, but may also be involved in periodontal bone destruction 1, 2, 3, 4, 5. Th17 cells are a subpopulation of T helper cells named after their signature cytokine, IL‐17A (henceforth called IL‐17) 6. IL‐17 may be a crucial mediator in protection against infection 7, 8. IL‐17 treatment of both immune and non‐immune cells induces the production of pro‐inflammatory cytokines such as IL‐6, chemokines and matrix metalloproteinases, leading to activation of innate immune cells, induction of pro‐inflammatory signalling pathways, and recruitment of neutrophils 9. IL‐17 has also been shown to be critical for immune homeostasis, particularly at mucosal barriers 10. In addition, there is increasing evidence of a critical role for IL‐17 and Th17 cells in the pathogenesis of inflammatory diseases such as psoriasis, psoriatic arthritis and ankylosing spondylitis (reviewed in 11).

In PD, there is evidence that IL‐17 may be protective against bacterial‐induced damage, but may also in some cases result in increased tissue damage. In a murine experimental PD model induced by the bacterium *Porphyromonas gingivalis* (*Pg*), KO of IL‐17RA resulted in markedly decreased neutrophil accumulation, causing accelerated periodontal bone loss 12. In support of this, surface‐glycan altered *Tannerella forsythia* has been shown to induce a Th17‐linked mobilization of neutrophils and decreased bone loss in a similar murine model of periodontal disease 13, 14.

In contrast, Eskan et al. have described a destructive role for IL‐17 in the pathogenesis of murine PD. In that study deficiency of the LFA‐1 integrin antagonist Del‐1 resulted in excessive neutrophil infiltration associated with increased periodontal bone loss. However, the bone destruction was prevented in Del‐1/IL‐17RA double‐deficient mice showing that neutrophil infiltration and increased bone loss were associated with IL‐17 signalling 15.

In addition to animal studies, emerging data show the presence of IL‐17 and Th17 cells in human PD 16, 17, 18, 19, 20. Recent studies show that *Pg* can promote an inflammatory IL‐17/Th17 response 21, 22, 23, but as yet little is known regarding the underlying mechanisms that drive and regulate an IL‐17/Th17 response in human PD.

In this study, we investigated how the periodontal pathogens *Pg* and *Aggregatibacter actinomycetemcomitans* (*Aa*; a periodontal pathogen particularly associated with human aggressive PD 24) affect IL‐17 production by human CD4^+^ T cells. Furthermore, we investigated whether there are quantitative or qualitative differences in IL‐17^+^ CD4^+^ T cells in the inflamed gingival tissue (GT) and peripheral blood from patients with PD when compared to healthy control (HC) subjects.

## Results

### *Pg* and *Aa* induce IL‐17 production in human monocyte/CD4^+^ T‐cell co‐cultures

**Figure 1:**
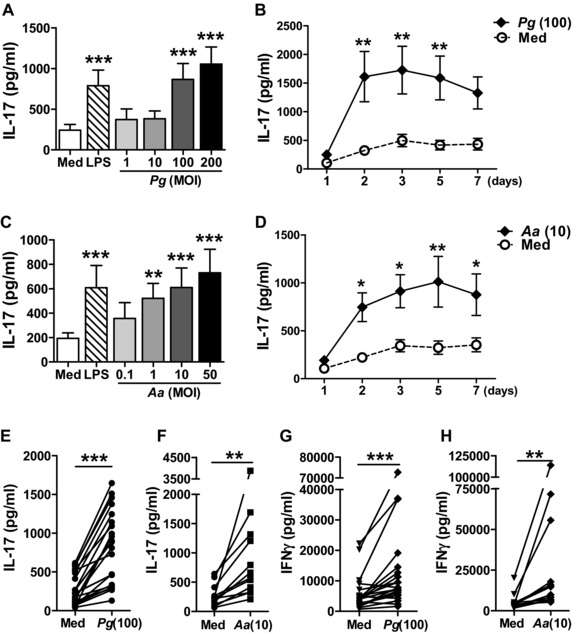
Heat‐killed *Pg* and *Aa* stimulate IL‐17 production in CD4^+^ T‐cell/monocyte co‐cultures. (A–D) CD4^+^ T cells (0.5 × 10^6^ cells) and CD14^+^ monocytes from PBMCs of healthy donors were co‐cultured at a 1:1 ratio in the presence of soluble anti‐CD3 mAbs without (Med) or with (A, B) heat‐killed *Pg* or (C, D) heat‐killed *Aa* (A, C) at the indicated MOI (multiplicity of infection) or (B, D) for different time periods. *Escherichia coli* LPS (100 ng/mL) was used as positive control. IL‐17 production in culture supernatants was measured by ELISA after 3 days (A, C, *n* = 6 different donors) or 1–7 days of culture (B, D, *n* = 8–9). Data are shown as the mean ± SEM. Data were analysed by one‐way (A, C) or two‐way (B, D) repeated measures ANOVA. Individual groups were compared to control (Med) values using the Bonferroni's multiple comparison test, *p < 0.05, **p < 0.01, ***p < 0.001. (E–H) Monocyte/CD4^+^ T‐cell co‐cultures were stimulated without (Med) or with (E, G) *Pg* (MOI = 100) or (F, H) *Aa* (MOI = 10) for 3 days. The production of (E, F) IL‐17 and (G, H) IFN‐γ in supernatants was measured by ELISA. Each symbol represents an individual donor (*n* = 12–22). Data were analysed by Wilcoxon matched‐pairs signed rank test. **p < 0.01, ***p < 0.001.

To investigate whether periodontal pathogens induce an IL‐17/Th17 response in vitro, CD4^+^ T cells and CD14^+^ monocytes were co‐cultured with anti‐CD3 mAbs in the presence or absence of either heat‐killed *Pg* strain W50 (*Pg*) or heat‐killed *Aa* strain Y4 (serotype b). Addition of *Pg* or *Aa* resulted in a dose‐ and time‐dependent induction of IL‐17 in co‐culture supernatants (Fig. 1A–F). In addition to IL‐17, we also detected an increase in IFN‐γ production in co‐culture supernatants following *Pg* or *Aa* stimulation (Fig. 1G and H).

### *Pg* and *Aa* activate human monocytes, leading to IL‐17 induction in CD4^+^ T cells

**Figure 2:**
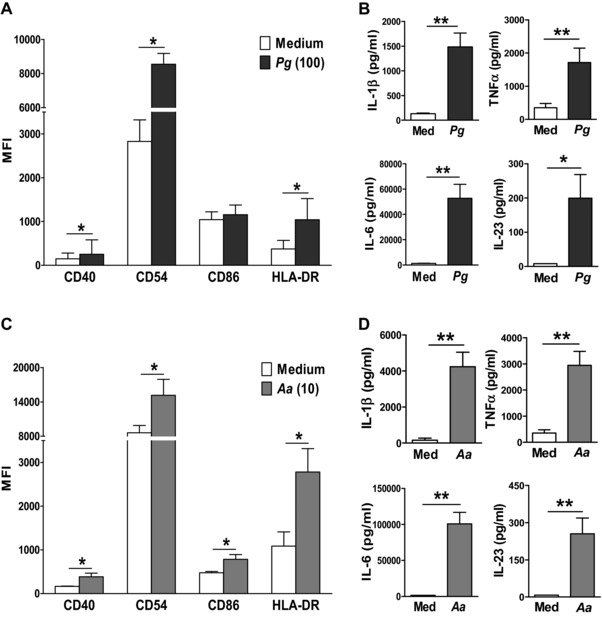
Periodontal pathogens activate CD14^+^ monocytes. (A, C) PB CD14^+^ monocytes from healthy control subjects were treated for 20 h with medium versus (A) *Pg* (MOI = 100) or (C) *Aa* (MOI = 10). Surface expression of the indicated markers was determined by flow cytometry. Data are shown as the median ± IQR (where IQR is interquartile range; *n* = 6 different donors). Log‐transformed data were analysed by paired *t*‐test. *p < 0.05. (B, D) Production of IL‐1β, TNF‐α, IL‐6 and IL‐23 by monocytes following 20 h stimulation with medium versus (B) *Pg* (MOI = 100, *n* = 8) or (D) *Aa* (MOI = 10, *n* = 8) was investigated by ELISA. Data are shown as the mean ± SEM. Log‐transformed data were analysed by paired *t*‐test, *p < 0.05, **p < 0.01.

To investigate the underlying mechanisms associated with *Pg‐* and *Aa*‐induced IL‐17 production in CD4^+^ T cells, we tested the effect of *Pg* or *Aa* stimulation on monocyte activation. Stimulation of monocytes with *Pg* resulted in significantly increased expression of the activation markers CD40, CD54 and HLA‐DR (Fig. 2A) and increased levels of IL‐1β, TNF‐α, IL‐6 and IL‐23 (Fig. 2B). Similar results were obtained for *Aa*‐stimulated monocytes, which also significantly upregulated CD86 expression (Fig. 2C and D). IL‐12p70 levels were very low and not significantly increased in *Pg*‐ or *Aa*‐stimulated monocytes (*n* = 9–10, data not shown). Also protein levels of IFN‐γ in cultures of *Pg‐* or *Aa*‐stimulated monocytes were negligible (*n* = 9–10, data not shown).

**Figure 3:**
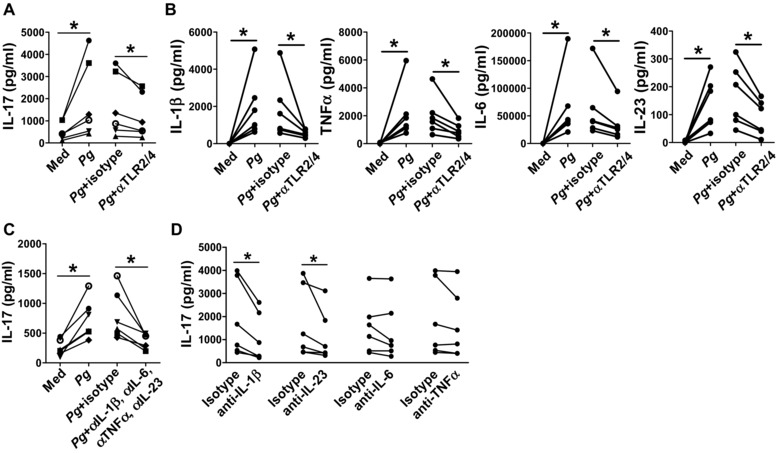
Mechanism underlying IL‐17 induction by *Pg*‐activated CD14^+^ monocytes. (A) CD4^+^ T cells (0.5 × 10^6^ cells) and CD14^+^ monocytes from PBMCs of healthy donors were co‐cultured for 3 days at a 1:1 ratio in the presence of soluble anti‐CD3 mAbs without (Med) or with *Pg* (MOI = 100) in the absence or presence of blocking antibodies to TLR2 and TLR4 or appropriate isotype control Abs (5 μg/mL). IL‐17 production in culture supernatants was measured by ELISA. (B) CD14^+^ monocytes (0.5 × 10^6^ cells) from the blood of healthy control subjects were incubated with medium or heat‐killed *Pg* (MOI = 100), in the absence or presence of blocking antibodies to TLR2 and TLR4, or appropriate isotype control Abs. Production of IL‐1β, TNF‐α, IL‐6 and IL‐23 in culture supernatants was investigated by ELISA. (C, D) CD4^+^ T cells (0.5 × 10^6^ cells) and CD14^+^ monocytes from PBMCs of healthy donors were co‐cultured for 3 days at a 1:1 ratio in the presence of soluble anti‐CD3 mAbs without (Med) or with *Pg* (MOI = 100) in the absence or presence of neutralising antibodies to IL‐1β, TNF‐α, IL‐6 and/or IL‐23 or the appropriate isotype control Abs. IL‐17 production in culture supernatants was measured by ELISA. Each symbol represents an individual donor (*n* = 6). Data were analysed by Wilcoxon matched‐pairs signed rank test, *p < 0.05.

TLR2 and TLR4 have been implicated in recognition of *Pg* by APCs 25, 26. Stimulation with *Pg* did not affect the expression of TLR2 or TLR4 (data not shown). Blocking both TLR2 and TLR4 significantly reduced the *Pg*‐induced IL‐17 production in supernatants from CD4^+^ T‐cell/monocyte co‐cultures (Fig. 3A). In cultures of purified CD14^+^ monocytes, TLR2 and TLR4 blockade decreased the secretion of IL‐1β, TNF‐α, IL‐6 and IL‐23 upon *Pg* stimulation (Fig. 3B), but did not affect the expression of the activation markers HLA‐DR, CD40, CD54 or CD86 (*n* = 4, data not shown). In cultures of purified CD4^+^ T cells, *Pg* addition did not lead to increased expression of the activation markers CD25, CD28 and CD69, or IL‐17 or IFN‐γ production (*n* = 4, data not shown). These data suggest that TLR2‐ and TLR4‐mediated recognition of *Pg* by monocytes contributes to subsequent Th17 polarisation. We tested the requirement for IL‐1β, TNF‐α, IL‐6 and IL‐23 in the *Pg*‐induced IL‐17/Th17 response by co‐culturing CD4^+^ T cells with monocytes, anti‐CD3 and *Pg* in the presence or absence of neutralising antibodies to IL‐1β, TNF‐α, IL‐6 and IL‐23. IL‐17 secretion was significantly reduced by adding a cocktail of the neutralising antibodies and by separately adding blocking antibodies to IL‐1β or IL‐23, whilst blockade of IL‐6 or TNF‐α did not significantly affect IL‐17 production by CD4^+^ T cells (Fig. 3C and D).

### Presence of IL‐17‐producing T cells in human GT

**Figure 4:**
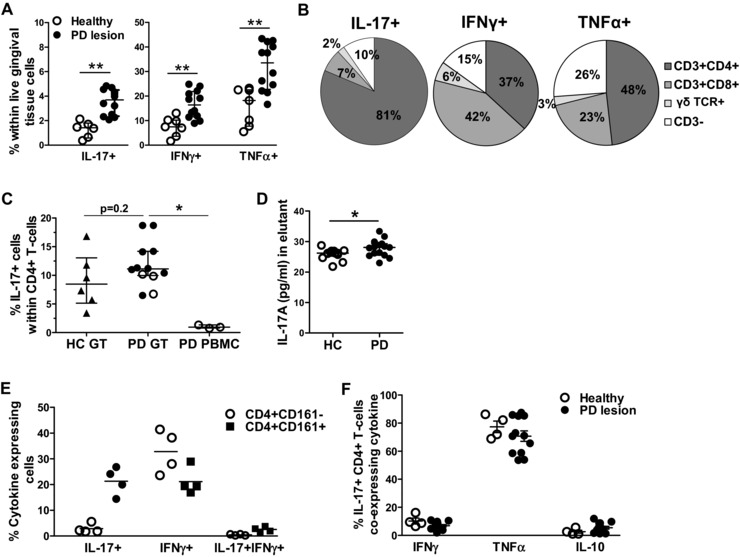
Presence and quantification of IL‐17^+^ CD4^+^ T cells in gingival tissue. Gingival tissue cells were isolated from inflamed gingival tissue samples from patients with periodontitis (PD lesion, *n* = 12) or from healthy gingival tissue samples from healthy donors (healthy, *n* = 6). Samples were digested with collagenase. Cells were stimulated ex vivo with PMA and ionomycin for 3 h prior to intracellular cytokine staining and flow cytometry. (A) Cumulative data showing the frequencies of IL‐17^+^ cells, IFN‐γ^+^ cells, and TNF‐α^+^ cells within FSC/SSC‐gated cells. Each symbol represents an individual donor sample and data are shown as scatter plots with median ± IQR (where IQR is interquartile range), analysed by unpaired *t*‐test of log‐transformed data, **p < 0.01; (B) Pie diagrams showing the average percentages of CD3^+^ CD4^+^ T cells, CD3^+^ CD8^+^ T cells, γδ T cells, or CD3^−^ cells within the IL‐17^+^ cells, IFN‐γ^+^ cells, and TNF‐α^+^ cells in gingival tissue cells from patients with periodontitis (mean of *n* = 3–12). (C) Cumulative data showing the percentages of CD4^+^ T cells expressing IL‐17 within gingival tissue from healthy controls (HC GT, *n* = 6) versus patients with periodontitis (PD GT, *n* = 12) versus PBMCs from patients with periodontitis (PD PBMCs, *n* = 3). Open circles indicate paired samples. Each symbol represents an individual donor sample and data are shown as median ± IQR, analysed by unpaired *t*‐test of log‐transformed data, *p < 0.05. (D) IL‐17 protein levels in GCF from patients with PD (*n* = 15) versus healthy controls (*n* = 13) were measured by Luminex assay. Minimal detection limit was 3.16 pg/mL. Data shown as mean ± SEM, analysed by unpaired *t*‐test, *p < 0.05. (E) Cumulative data showing the percentages of IL‐17^+^ and IFN‐γ^+^ cells within CD161^+^ or CD161^−^ CD4^+^ T cells in diseased gingival tissue from patients with periodontitis (*n* = 4). Each symbol represents an individual donor, lines show the mean. (F) Cumulative data showing the percentages of IL‐17^+^ CD4^+^ T cells that co‐express IFN‐γ, TNF‐α or IL‐10 in gingival tissue from patients with periodontitis (*n* = 12) versus healthy controls (*n* = 4). Each symbol represents an individual donor, lines show the mean ± SEM.

Next, we investigated the presence of IL‐17‐producing cells in inflamed GT from PD lesions. GT cells were isolated from diseased and healthy GT samples by collagenase digestion. When gated on total viable single cells (gating strategy shown in Supporting Information Fig. 1), increased proportions of IL‐17^+^, IFN‐γ^+^ and TNF‐α^+^ cells were seen in PD lesions (Fig. 4A). Using back‐gating (gating strategy shown in Supporting Information Fig. 2), the IL‐17^+^ cells in diseased tissue were found to be predominantly CD3^+^ CD4^+^ T cells (on average 81%), with on average 7 and 10% of the IL‐17^+^ cells being CD8^+^ T cells and CD3^−^ cells, respectively, and a small percentage (∼2%) of IL‐17^+^ cells being γδ T cells (Fig. 4B). In contrast, IFN‐γ^+^ and TNF‐α^+^ cells comprised on average 37% and 48% of CD4^+^ T cells, respectively, with the remaining proportion being predominantly CD3^+^ CD8^+^ cells and CD3^−^ cells (Fig. 4B).

We then quantified the frequencies of IL‐17‐producing CD4^+^ T cells within GT cells from diseased lesions relative to healthy GT, and for a small number of cases, also paired PBMCs from patients with PD (gating strategy shown in Supporting Information Fig. 3). Approximately 12% (median, range 5–18%) of CD4^+^ T cells expressed IL‐17 in periodontal lesions, which was slightly higher compared to the frequency in healthy GT (median 9%, range 3–16%, *p* = 0.2), and significantly higher than the percentage seen in the blood of patients with PD (median 0.9%, range 0.8–1.3%, *p* = 0.01; Fig. 4C). On average, the percentages of IL‐17^+^ cells within the CD8^+^ and γδ T‐cell populations were 1.4 ± 0.2 and 4.1 ± 2.1%, respectively (Supporting Information Fig. 3). IL‐17 protein was also detectable in gingival crevicular fluid (GCF) from patients with PD at slightly higher levels compared to GCF from healthy subjects (*p* = 0.03, Fig. 4D).

It has been proposed that human IL‐17‐producing Th cells originate from a CD161^+^ CD4^+^ T‐cell precursor 27. We determined the presence of IL‐17‐ or IFN‐γ‐expressing cells in CD161^+^ versus CD161^−^ CD4^+^ T cells (gating strategy shown in Supporting Information Fig. 4). An enrichment of IL‐17‐expressing cells within CD161^+^ CD4^+^ T cells was observed when compared to the CD161^−^ CD4^+^ T cells, whereas similar percentages of IFN‐γ‐expressing CD4^+^ T cells were found within CD161^+^ and CD161^−^ cells (Fig. 4E). In addition, a slight increase in the percentage of cells that express both these cytokines was observed in CD161^+^ versus CD161^−^ CD4^+^ T cells.

Recent data indicate that different subsets of Th17 cells may exist producing either IFN‐γ or IL‐10 28, 29, 30. We therefore determined the co‐expression of inflammatory (IFN‐γ, TNF‐α) and anti‐inflammatory (IL‐10) cytokines by IL‐17^+^ CD4^+^ T cells in healthy versus inflamed GT. In PD lesions, on average 7 ± 1% of the IL‐17^+^ CD4^+^ T cells co‐expressed IFN‐γ, 7 ± 1% co‐expressed IL‐10, and a high proportion co‐expressed TNF‐α (on average 69 ± 4%) however this was not significantly different from healthy GT (Fig. 4F, gating strategy shown in Supporting Information Fig. 5).

### CD4^+^ T cells from patients with PD show an enhanced IL‐17 response

**Figure 5:**
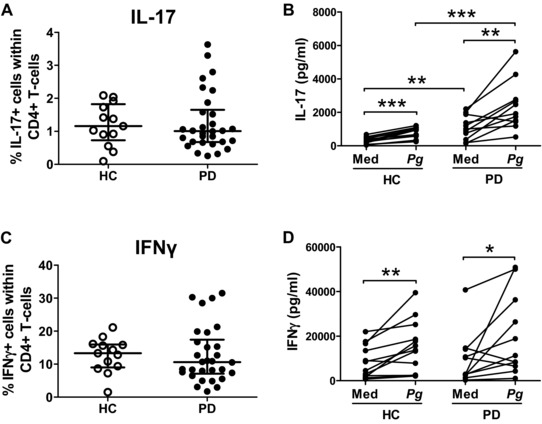
Peripheral CD4^+^ T cells from patients with periodontitis show an enhanced IL‐17 response upon stimulation. (A, C) PBMCs were isolated from patients with periodontitis (PD, *n* = 30) or from periodontally healthy controls (HC, *n* = 13). Cells were stimulated ex vivo with PMA and ionomycin for 3 h prior to intracellular staining. Data show the percentage of (A) IL‐17^+^ or (C) IFN‐γ^+^ cells within CD4^+^ T cells. Each symbol represents an individual donor, lines show the median ± IQR (where IQR is interquartile range). Log‐transformed data were analysed by unpaired *t*‐test. (B, D) CD4^+^ T cells (0.5 × 10^6^ cells) and CD14^+^ monocytes were isolated from PBMCs of periodontally healthy donors (*n* = 13) and periodontitis patients (*n* = 11), and co‐cultured at a 1:1 ratio in the presence of soluble anti‐CD3 mAbs with or without heat‐killed *Pg* (MOI = 100) for 7 days. Production of (B) IL‐17 or (D) IFN‐γ production in culture supernatants was measured by ELISA. Each symbol represents an individual donor. Log‐transformed data were analysed by paired and unpaired *t*‐test, *p < 0.05, **p < 0.01, ***p < 0.001.

Finally, we investigated whether the percentage of IL‐17^+^ CD4^+^ T cells was increased in peripheral blood from patients with PD relative to periodontally HCs. For this purpose we collected PBMCs from patients with PD (*n* = 15 chronic PD and *n* = 15 aggressive PD) and HC (*n* = 13, gender and age matched; see Supporting Information Table 1). Cells were cryopreserved and then analysed in batches of five different samples (consisting of a mix of HC and PD) for ex vivo cytokine expression. No significant difference was found in the percentage of IL‐17^+^ or IFN‐γ^+^ cells within CD4^+^ T cells in PBMCs between patients with PD and HCs (Fig. 5A and C). However, higher IL‐17 protein levels were detected in co‐cultures of monocytes and CD4^+^ T cells from patients with PD, and this was significantly enhanced further upon *Pg* stimulation (Fig. 5B). IFN‐γ production, in contrast, was not significantly different between HCs and patients with PD (Fig. 5D).

Both IL‐17 and IFN‐γ were produced at increased levels when CD4^+^ T cells were cultured with monocytes in the presence of *Pg* or *Aa* alone (i.e. in the absence of anti‐CD3 mAbs). This was observed using cells derived from HCs as well as PD patients (Supporting Information Fig. 6). These data highlight the possibility that the IL‐17 and IFN‐γ production by the CD4^+^ T cells could be antigen specific; however we cannot exclude the possibility that this is due to bystander activation caused by innate immune activation due to TLR stimulation. Further work is required to confirm this.

## Discussion

In this study, we investigated the role of the periodontal pathogens *Pg* and *Aa* in the induction of a Th17/IL‐17 response in periodontal disease. Our data show that the periodontal pathogens *Pg* and *Aa* activate monocytes, which can subsequently induce an IL‐17/Th17 response in CD4^+^ T cells in vitro. We demonstrate that the underlying mechanism involves the recognition of *Pg* by TLR2/4 on monocytes leading to an IL‐1β/IL‐23‐dependent induction of IL‐17 production by CD4^+^ T cells. IL‐1β and IL‐23 have previously been reported to be required to induce the differentiation or generation of human Th17 cells from naïve CD4^+^ T cells or effector memory CD4^+^ T cells 27, 31, and our data are consistent with those studies. We also show that IL‐17^+^ CD4^+^ T cells and IL‐17 protein are clearly detectable in diseased as well as healthy GT and GCF. Furthermore, our data suggest that although there is no increase in the frequency of IL‐17^+^ CD4^+^ T cells in the peripheral blood of patients with PD, peripheral CD4^+^ T cells from these patients show enhanced IL‐17 production in response to *Pg* stimulation.

Previous studies have shown that monocyte‐derived DCs primed with different *Pg* strains elicited distinct T‐cell responses. Specifically it has been shown that certain capsular strains of *Pg* (W50, HG184) were capable of inducing a Th1/Th17 response when compared with the non‐encapsulated strains or other capsular serotypes of *Pg* 21, 22. In our study, we used *Pg* capsular strain W50 and *Aa* strain Y4 (serotype b) and found these pathogens induced IL‐17 production by CD4^+^ T cells when co‐cultured with monocytes. *Aa* appears to be more potent than *Pg* in this in vitro system, as it induced IL‐17 production and monocyte activation at lower multiplicity of infection (MOI). These in vitro data are in line with data from a recent mouse study that showed that *Pg* stimulation induced a TLR2‐ and IL‐1‐driven Th17/IL‐17 response by splenic CD4^+^ T cells co‐cultured with splenic APCs 32. Interestingly, in that study *Pg*‐infection induced not only PD but also directly promoted autoimmune experimental arthritis, which correlated with the *Pg*‐induced collagen‐specific Th17 response in mice 32.

In addition, a recently published paper suggested that the periodontal bone loss observed in patients with leukocyte adhesion deficiency type I (LAD‐I) is associated with an abundant infiltration of IL‐17‐producing CD3^+^ T cells and the excessive production of IL‐17 in GT 33. The authors further showed that IL‐17 overproduction in untreated LFA‐1^KO^ mice (a model of LAD‐I PD) was dependent on IL‐23 and that local treatment with antibodies to IL‐17 or IL‐23 in LFA‐1^KO^ mice blocked inflammatory periodontal bone loss. Although, LAD‐I‐associated PD is a very rare and specific subtype of PD, it is of interest that our in vitro data indicate that patients with PD may also have an exaggerated IL‐17 production upon stimulation. Together these data support the notion that the IL‐23/IL‐17 axis may play an important role in the biology of PD 18, 19, 34.

The ability of periodontal pathogen‐stimulated APCs to produce IL‐6, IL‐1β, TNF‐α and IL‐23 may contribute to Th17 induction in situ 18. Indeed, an increased percentage of IL‐17‐producing cells were detected in diseased GT. These results are in line with data on IL‐17‐expressing CD3^+^ or CD4^+^ T cells obtained using immunohistochemistry 17, 35, 36. We further detected that these IL‐17‐producing cells were predominantly CD4^+^ T cells, and were enriched in the CD161^+^ fraction of CD4^+^ T cells. We also observed that IFN‐γ‐expressing cells were found within CD161^+^ CD4^+^ T cells. These data may indicate that some of the IL‐17^+^ cells have become Th1‐like as was previously suggested 27, but further work is required to confirm the origin of the Th1 and Th17 cells in GT.

Although there is evidence that IL‐17 production by gingival γδ T cells may play a potential role in tissue damage in experimental PD 15, in our study only very few of the IL‐17^+^ cells were γδ T cells. We previously showed that IL‐17^+^ CD8^+^ T cells are enriched in the joints of patients with psoriatic arthritis and correlate with clinical parameters of active disease 37. However, only a low percentage of IL‐17^+^ cells in diseased GT were found to be CD8^+^ T cells. These findings suggest that the infiltration of IL‐17‐producing CD4^+^ T cells may be important in relation to the periodontal bone destruction in inflamed GT. It should be noted that we also detected increased levels of IFN‐γ‐ and TNF‐α‐expressing cells in diseased GT, suggesting an inflammatory cell infiltrate and these cells may also contribute to immunopathology in PD.

One possible explanation for the presence of IL‐17^+^ CD4^+^ T cells in both PD lesions and healthy GT could be the existence of pathogenic and non‐pathogenic Th17 cell subsets. In a mouse model, Th17 cells generated with TGF‐β1 and IL‐6 produce IL‐17 but do not readily induce autoimmune disease without further exposure to IL‐23, whilst Th17 generated in the presence of IL‐23 or TGF‐β3 and IL‐6 induced severe EAE 28, 38, 39, 40. A recent study using human T cells showed that different subsets of Th17 cells evolve in response to different types of pathogens 29, 41. Additionally, IL‐17‐producing CD4^+^ T cells can co‐express the anti‐inflammatory cytokine IL‐10 upon modulation of the inflammatory milieu by TNF blockade 30. However, our analysis did not reveal a significant difference between patients with PD and periodontally HCs in the percentage of gingival IL‐17^+^ CD4^+^ T cells co‐expressing TNF‐α, IFN‐γ or IL‐10.

In conclusion, our data provide evidence that *Pg* and *Aa* can activate monocytes resulting in increased IL‐17 production by human CD4^+^ T cells in vitro, a process that appears enhanced in patients with PD.

## Materials and methods

### Patient and HC samples

HC samples were obtained from periodontally healthy volunteers. Patients with PD were recruited from the periodontal clinic at Guy's Hospital (Supporting Information Table 1). GT samples from patients with PD were obtained at periodontal surgery or from periodontally HC subjects undergoing non‐periodontal disease‐related procedures (e.g. crown lengthening or tooth extraction) in the periodontal department at Guy's Hospital. Oral and periodontal examination records, age, sex and smoking status were documented on the day of sample collection. Periodontal disease case definitions for moderate to severe chronic PD were used according to those described by Page and Eke, and for aggressive PD according to those proposed by Demmer and Papapanou 42, 43, with the presence of two or more interproximal, nonadjacent sites with attachment loss of ≥6 mm occurring at a minimum of two different teeth and accompanied by bleeding on probing. All clinical investigations were conducted according to the Declaration of Helsinki principles. Ethics approval for this study was given by the NRES Committee London ‐ City Road & Hampstead Research Ethics Committee (12/LO/0376). Written informed consent was received from participants before their inclusion in the study.

### Cell isolation from peripheral blood

PBMCs were isolated by density gradient centrifugation using lymphocyte separation medium (LSM 1077; PAA Laboratories, Pasching, Austria or Lymphoprep; AXIS‐SHIELD, Oslo, Norway). PBMCs were used freshly or cryopreserved within 1 h of isolation and stored in liquid nitrogen in medium containing 90% foetal bovine serum (lot 030M3399, Sigma‐Aldrich, St. Louis, MO, USA) and 10% dimethyl sulfoxide (Sigma‐Aldrich). CD14^+^ monocyte and CD4^+^ T‐cell isolation from PBMCs was performed by magnetic cell sorting (Miltenyi Biotec, Bergisch‐Gladbach, Germany) via positive and negative selection, respectively. Purity was confirmed by flow cytometry and was consistently found to be >96% for monocytes and >95% for CD4^+^ T cells.

### Cell isolation from GT

GT collected from surgical procedures was weighed and cut into approximately 1 mm^3^ fragments and digested using 2 mg/mL collagenase type 1 (Worthington Biochemical Corporation, Lakewood, NJ, USA) at 37°C for 1 h. The ratio of tissue to medium was 25 mg to 1 mL. The residual fragments of tissue were dissociated by gentle flushing with a pipette and then filtered through cell strainers of mesh size 70 μm. Disaggregated cell fractions were washed and resuspended in culture medium followed by ex vivo stimulation and staining for cytometry.

### Collection of GCF

GCF samples were collected from three diseased sites (probing depth ≥5 mm) from patients with PD and from three healthy sites (probing depth ≤3 mm, no bleeding on probing) from periodontally HC subjects. The tooth for sampling was isolated with a cotton wool roll and the supragingival plaque was gently removed with a cotton wool roll before sample acquisition. GCF samples were collected using strips (2 mm × 8 mm) of Durapore^®^ polyvinylidene difluoride hydrophilic membrane filters (pore size of 0.22 μm, GVWP04700; Millipore, Watford, UK) that were gently placed into the periodontal crevice until mild resistance was detected, and left in place for 30 s. Strips contaminated by blood were discarded. After GCF collection, each strip was placed in an Eppendorf tube containing 150 μL of PBS (Sigma, UK) on ice. The Eppendorf tubes were vortexed for 10 s and then left at 4°C for 15 min to elute GCF from the strip and vortexed again. The eluted solution was centrifuged at 10 000 rpm for 15 min at 4°C before removing the strips, and stored at −80°C until further analysis. Three tubes of eluted solution of healthy sites from a healthy subject or three tubes of eluted solution of diseased sites from a patient with PD were pooled and mixed well prior to assays.

### Ex vivo cytokine staining

PBMCs and disaggregated GT cells were stimulated for 3 h with PMA (50 ng/mL) and ionomycin (750 ng/mL; both purchased from Sigma‐Aldrich) in the presence of GolgiStop (BD, Oxford, UK), according to the manufacturer's instructions. Extracellular surface staining was performed using PE‐Cy7‐conjugated anti‐CD3 (clone UCHT1), APC‐Cy7‐conjugated anti‐CD14 (clone HCD14), Brilliant Violet 605‐conjugated anti‐CD19 (clone HIB19; all purchased from BioLegend, London, UK), PE‐CF594‐conjugated anti‐CD8 (clone RPA‐T8), FITC‐conjugated anti‐γδ TCR (clone 11F2; both purchased from BD Biosciences, Oxford, UK). The cells were fixed with 2% paraformaldehyde and permeabilized with 0.5% saponin (Sigma) and stained intracellularly with Pacific Blue‐conjugated anti‐CD4 (clone SK3), Alexa Fluor 488‐conjugated anti‐IL‐10 (clone JES3‐9D7), PE‐conjugated anti‐IL‐17A (clone BL168), APC‐conjugated anti‐TNF‐α (clone MAb11), and PerCP‐Cy5.5‐conjugated anti‐IFN‐γ (clone 4S.B3; all purchased from BioLegend). Cells were acquired using a FACSCanto or LSRFortessa (BD Biosciences, Franklin Lakes, NJ, USA) and analysed using the FlowJo software (Tree Star, Ashland, OR, USA). Cytometer settings were tracked over time using BD Biosciences Cytometer Setup and Tracking software to monitor cytometer performance. Viable single cells were identified using a LIVE/DEAD^®^ Fixable Blue Dead Cell stain kit (Life technologies, Carlsbad, CA, USA) or based on their FSC‐A/FSC‐W profile.

### Bacteria

Heat‐killed bacteria were kindly provided by Prof. Mike Curtis (Barts and The London School of Medicine and Dentistry, Queen Mary University of London). *Pg* strain W50 was grown at 37°C in brain heart infusion broth supplemented with hemin (1 μg/mL) in an anaerobic atmosphere of 80% N_2_, 10% H_2_, and 10% CO_2_ (Don Whitley Scientific) for 48 h. *Aa* strain Y4 was cultured in brain heart infusion medium supplemented with 0.05% cysteine and 0.5% yeast extract and incubated in anaerobic conditions for 48 h. Cells were harvested by centrifugation at 10 000 × *g* for 15 min at 4°C. Cell pellets were suspended and washed in PBS and then resuspended in PBS to an OD of 1.0 at 600 nm. The bacteria were then heat‐killed at 65°C for 60 min, washed and resuspended in PBS at a final density of 5 × 10^9^ CFU/mL. Non‐viability was confirmed by plating aliquots and incubating as described above.

### Cell culture

CD4^+^ T cells (0.5 × 10^6^ cells) and CD14^+^ monocytes were co‐cultured at a 1:1 ratio with αCD3 mAbs (Okt‐3, Janssen‐Cilag, Buckinghamshire, UK) in culture medium in a 24‐well plate in the presence or absence of heat‐killed *Pg* or *Aa* at the indicated MOI for different time points. *Escherichia coli* LPS (100 ng/mL, Sigma) was used as a positive control. In monocyte only cultures, cells (0.5 × 10^6^/mL) were stimulated with *Pg* or *Aa* at the indicated MOI for 20 h. Neutralising mAbs to IL‐1β (clone 8516, mIgG1), IL‐6 (clone 1936, mIgG2b), TNF‐α (clone 6401, mIgG1; all purchased from R&D Systems Europe Limited, Abingdon, UK), IL‐23p19 (clone HNU2319; eBiosciences, San Diego, CA, USA) or isotype controls (mIgG1, clone 11711, mIgG2a, clone 20102, mIgG2b, clone 20116 and hIgG1 Fc; R&D Systems Europe Limited) were used at 10 μg/mL. Anti‐TLR‐2 (clone TL2.1) and TLR‐4 (clone HTA125) mAbs (BioLegend) were used at 5 μg/mL.

### Analysis of monocyte phenotype

CD14^+^ monocyte phenotype was assessed after 20 h of culture with bacteria, by staining with APC‐Cy7‐conjugated anti‐CD14 (clone HCD14), APC‐conjugated anti‐CD54 (clone HCD54), Pacific Blue‐conjugated anti‐CD86 (clone IT2.2; all purchased from BioLegend), PE‐conjugated anti‐CD40 (clone LOB7/6, AbD Serotec, Kidlington, UK), PerCP‐Cy5.5‐conjugated anti‐HLA‐DR (clone G46‐6, BD Biosciences, Oxford, UK), FITC‐conjugated anti‐TLR2 (clone TL2.1), PE‐conjugated anti‐TLR4 (clone HTA125; both purchased from eBiosciences).

### Cytokine detection

Culture supernatants were collected and stored at −80°C until further use. The levels of IL‐23 (eBioscience), IL‐17, IL‐1β, IL‐6, IFN‐γ, IL‐12p70 and TNF‐α (all purchased from BioLegend) were detected using ELISA according to the manufacturers’ protocols. IL‐17A levels in GCF were detected by Luminex assay (Bio‐Plex Pro^TM^ Human Th17 Cytokine Assays, Bio‐Rad Laboratories, CA, USA).

### Statistical analysis

Values are expressed as mean with SEM or as median with interquartile range. Data were tested for normality using D'Agostino & Pearson omnibus normality testing and where not normally distributed data were log10 transformed prior to analysis (which provided normally distributed results). Comparisons between patients and HCs were made using one‐way ANOVA followed by Bonferroni's multiple comparison test for parametric data. Data were analysed using Prism version 5 (GraphPad Software Inc., La Jolla, USA). For all tests, *p* values of less than 0.05 were considered significant.

## Conflict of interest

L.S.T. has received speaker fees and research funding from UCB, Novo Nordisk A/S, GSK and Novartis AG. All other authors declare no financial or commercial conflict of interest.

**Abbreviations**
*Aa*: *Aggregatibacter actinomycetemcomitans*
GCF: gingival crevicular fluid
GT: gingival tissue
HC: healthy control
LAD‐I: leukocyte adhesion deficiency type I
MOI: multiplicity of infection
PD: periodontitis
*Pg*: *Porphyromonas gingivalis*

## Supporting information

As a service to our authors and readers, this journal provides supporting information supplied by the authors. Such materials are peer reviewed and may be re‐organized for online delivery, but are not copy‐edited or typeset. Technical support issues arising from supporting information (other than missing files) should be addressed to the authors.

**Supplementary Figure 1**. Gating strategy to identify IL‐17, IFNγ or TNFα producing cells ingingival tissue. Gingival tissue cells were isolated from an inflamed gingival tissue sample from a patient with periodontitis. Samples were digested with collagenase. Cells were stimulated ex vivo with PMA and ionomycin for 3 h prior to intracellular cytokine staining. The following gating strategy was employed: first, the live lymphocyte population was gated (A), and then doublets were excluded based on FSC‐W versus FSC‐A (B). Total IL‐17+ or IFNγ+ or TNFα+ cells were gated using the cell gates shown (C‐E), which were based on negative control stains (FMO; F‐H).
**Supplementary Figure 2**. Gating strategy to identify IL‐17, IFNγ or TNFα producing CD4+, CD8+ or γδ T‐cells in gingival tissue. Gingival tissue cells were isolated from an inflamed gingival tissue sample from a patient with periodontitis. Samples were digested with collagenase. Cells were stimulated ex vivo with PMA and ionomycin for 3 h prior to intracellular cytokine staining. A gate was set on cells that stained positive for the cytokines IL‐17 (A), IFNγ (B) or TNFα (C), and subsequently the percentages of CD3+, CD3+CD4+, CD3+CD8+ and CD3+γδ+ cells within these gates were determined. Representative gating strategy and typical dot plots for the CD3+CD4+, CD3+CD8+ or γδ T‐cells within the cytokine‐positive cells are shown.
**Supplementary Figure 3**. Quantification of IL‐17+, IFNγ+ or TNFα+ cells within CD4+, CD8+ or γδ T‐cells in gingival tissue. Gingival tissue cells were isolated from inflamed gingival tissue samples from patients with periodontitis (*n* = 3–8). Samples were digested with collagenase. Cells were stimulated ex vivo with PMA and ionomycin for 3 h prior to intracellular cytokine staining. (A) Representative gating strategy and (B‐D) cumulative data showing the percentages of IL‐17+, IFNγ+ or TNFα+ cells within CD4+ T‐cells (B), CD8+ T‐cells (C) or γδ T‐cells (D).
**Supplementary Figure 4**. Quantification of IL‐17+ and IFNγ+ cells within CD4+CD161+ Tcells in gingival tissue. Gingival tissue cells were isolated from inflamed gingival tissue samples from patients with periodontitis (n=4). Samples were digested with collagenase. Cells were stimulated *ex vivo* with PMA and ionomycin for 3 h prior to intracellular cytokine staining. Representative gating strategy to identify CD161+ versus CD161‐ CD4+ T‐cells and the percentages of IL‐17+ and IFNγ+ cells within these populations
