# Supplementary material for: Periodontitis‐associated pathogens P. gingivalis and A. actinomycetemcomitans activate human CD14+ monocytes leading to enhanced Th17/IL‐17 responses
Source: Eur J Immunol. 2016 Aug 1;46(9):2211–21. doi: 10.1002/eji.201545871 (PMC5031191; doi:10.1002/eji.201545871)
Supplement: Supplementary file 1 — Supplementary Figure 1. Gating strategy to identify IL‐17, IFNγ or TNFα producing cells ingingival tissue. Gingival tissue cells were isolated from an inflamed gingival tissue sample from a patient with periodontitis. Samples were digested with collagenase. Cells were stimulated ex vivo with PMA and ionomycin for 3 h prior to intracellular cytokine staining. The following gating strategy was employed: first, the live lymphocyte population was gated (A), and then doublets were excluded based on FSC‐W versus FSC‐A (B). Total IL‐17+ or IFNγ+ or TNFα+ cells were gated using the cell gates shown (C‐E), which were based on negative control stains (FMO; F‐H). Supplementary Figure 2. Gating strategy to identify IL‐17, IFNγ or TNFα producing CD4+, CD8+ or γδ T‐cells in gingival tissue. Gingival tissue cells were isolated from an inflamed gingival tissue sample from a patient with periodontitis. Samples were digested with collagenase. Cells were stimulated ex vivo with PMA and ionomycin for 3 h prior to intracellular cytokine staining. A gate was set on cells that stained positive for the cytokines IL‐17 (A), IFNγ (B) or TNFα (C), and subsequently the percentages of CD3+, CD3+CD4+, CD3+CD8+ and CD3+γδ+ cells within these gates were determined. Representative gating strategy and typical dot plots for the CD3+CD4+, CD3+CD8+ or γδ T‐cells within the cytokine‐positive cells are shown. Supplementary Figure 3. Quantification of IL‐17+, IFNγ+ or TNFα+ cells within CD4+, CD8+ or γδ T‐cells in gingival tissue. Gingival tissue cells were isolated from inflamed gingival tissue samples from patients with periodontitis (n = 3–8). Samples were digested with collagenase. Cells were stimulated ex vivo with PMA and ionomycin for 3 h prior to intracellular cytokine staining. (A) Representative gating strategy and (B‐D) cumulative data showing the percentages of IL‐17+, IFNγ+ or TNFα+ cells within CD4+ T‐cells (B), CD8+ T‐cells (C) or γδ T‐cells (D). Supplementary Figure 4. Quantification of IL [file EJI-46-2211-s001.pdf]

European Journal of Immunology

Supporting Information
for
DOI 10.1002/eji.201545871

Wan-Chien Cheng, Saskia D. van Asten, Lachrissa A. Burns, Hayley G. Evans, Gina J. Walter, Ahmed Hashim, Francis J. Hughes and Leonie S. Taams

Periodontitis-associated pathogens *P. gingivalis* and *A. actinomycetemcomitans* activate human CD14^{+} monocytes leading to enhanced Th17/IL-17 responses

© 2016 The Authors. *European Journal of Immunology* published by WILEY-VCH Verlag GmbH & Co. KGaA, Weinheim.

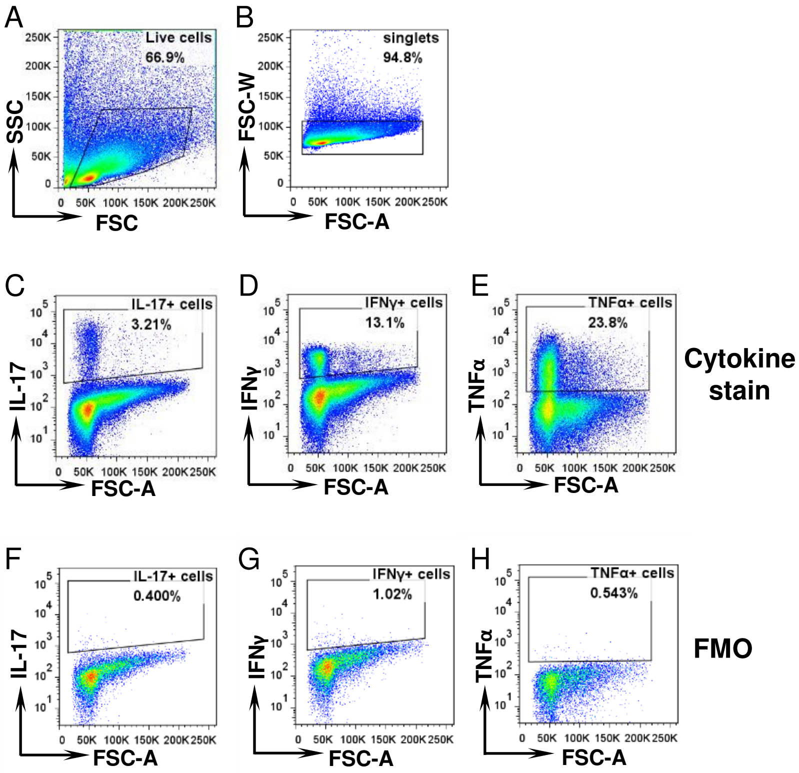

Supplementary Figure 1. Gating strategy to identify IL-17, IFNγ or TNFα producing cells in gingival tissue. Gingival tissue cells were isolated from an inflamed gingival tissue sample from a patient with periodontitis. Samples were digested with collagenase. Cells were stimulated ex vivo with PMA and ionomycin for 3 hours prior to intracellular cytokine staining. The following gating strategy was employed: first, the live lymphocyte population was gated (A), and then doublets were excluded based on FSC-W vs. FSC-A (B). Total IL-17+ or IFNγ+ or TNFα+ cells were gated using the cell gates shown (C-E), which were based on negative control stains (FMO) (F-H).

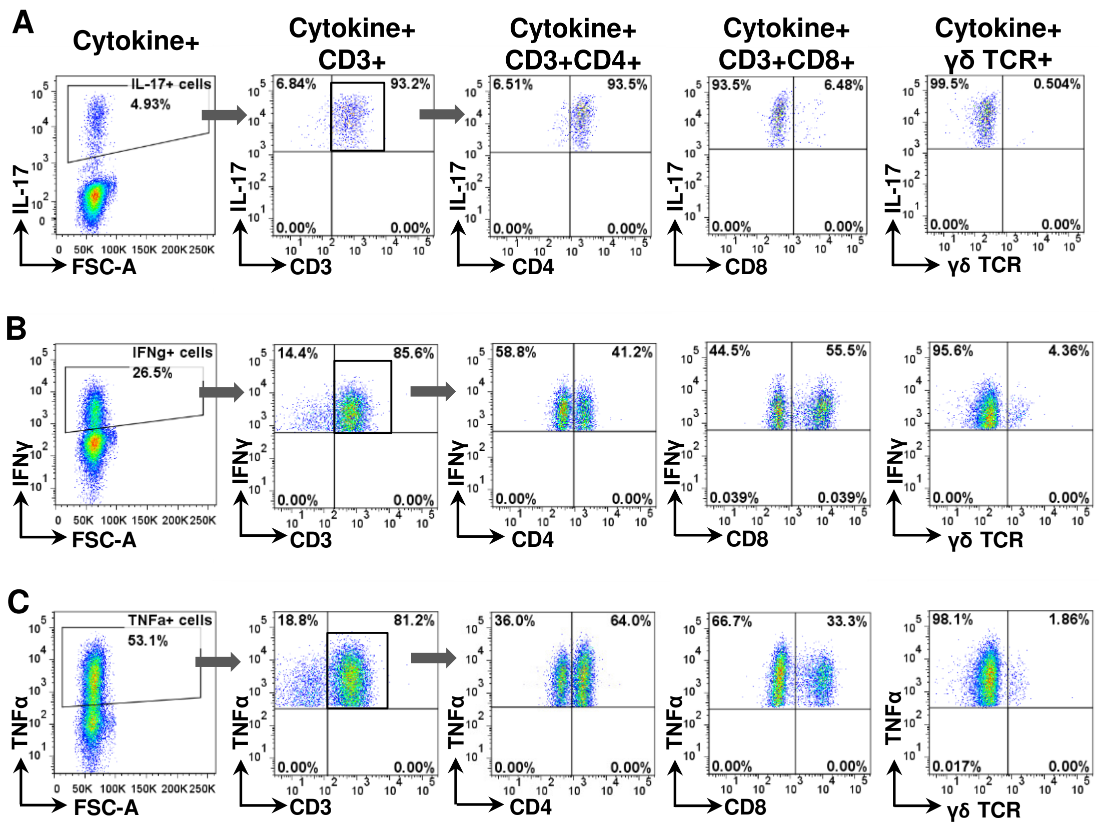

Supplementary Figure 2. Gating strategy to identify IL-17, IFNγ or TNFα producing CD4+, CD8+ or γδ T-cells in gingival tissue. Gingival tissue cells were isolated from an inflamed gingival tissue sample from a patient with periodontitis. Samples were digested with collagenase. Cells were stimulated ex vivo with PMA and ionomycin for 3 hours prior to intracellular cytokine staining. A gate was set on cells that stained positive for the cytokines IL-17 (A), IFNγ (B) or TNFα (C), and subsequently the percentages of CD3+, CD3+CD4+, CD3+CD8+ and CD3+γδ+ cells within these gates were determined. Representative gating strategy and typical dot plots for the CD3+CD4+, CD3+CD8+ or γδ T-cells within the cytokine-positive cells are shown.

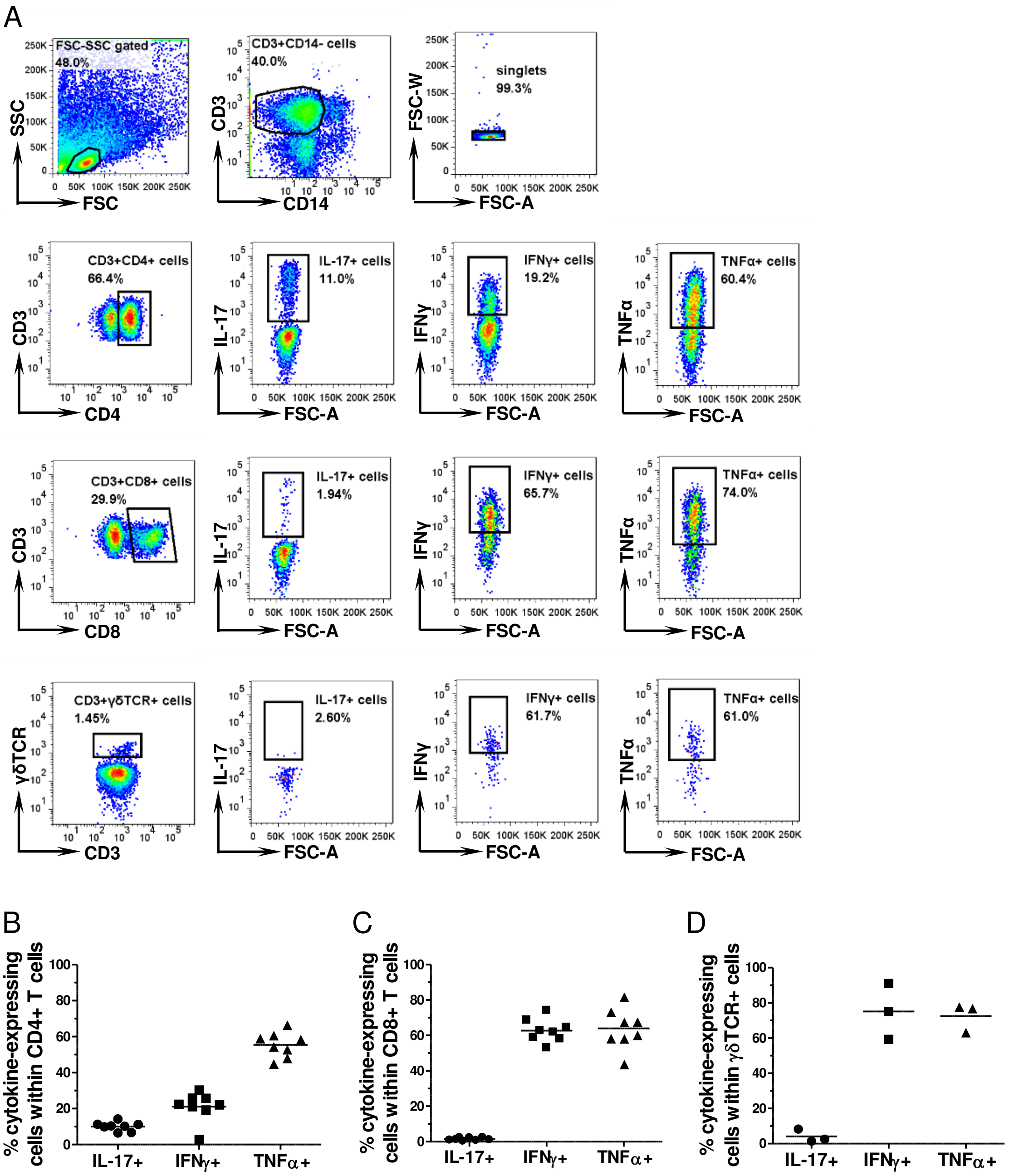

Supplementary Figure 3. Quantification of IL-17+, IFNγ+ or TNFα+ cells within CD4+, CD8+ or γδ T-cells in gingival tissue. Gingival tissue cells were isolated from inflamed gingival tissue samples from patients with periodontitis (n=3-8). Samples were digested with collagenase. Cells were stimulated ex vivo with PMA and ionomycin for 3 hours prior to intracellular cytokine staining. (A) Representative gating strategy and (B-D) cumulative data showing the percentages of IL-17+, IFNγ+ or TNFα+ cells within CD4+ T-cells (B), CD8+ T-cells (C) or γδ T-cells (D).

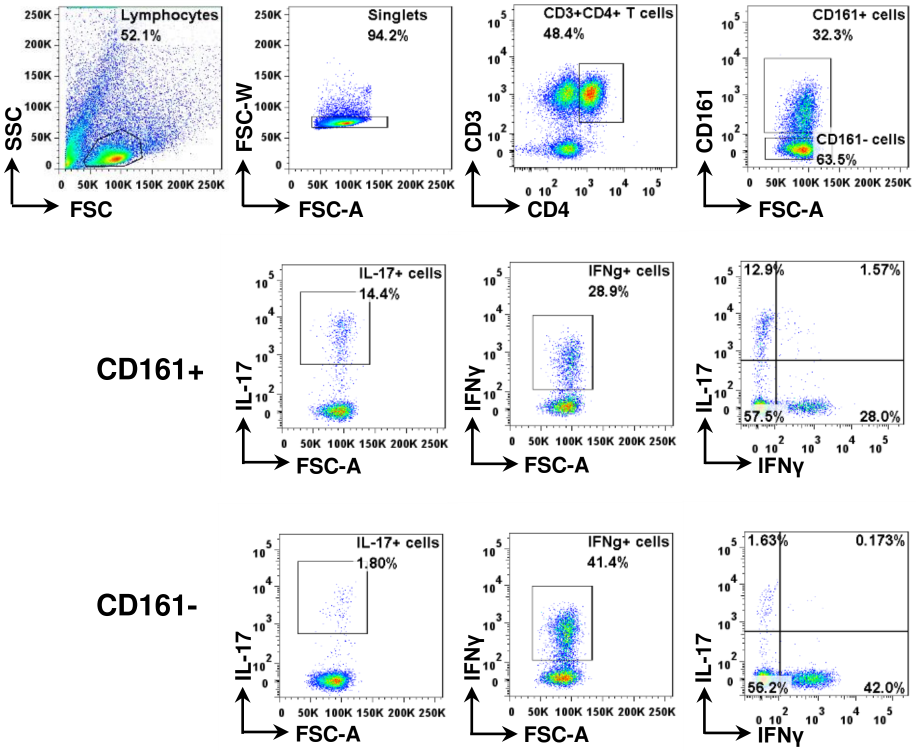

Supplementary Figure 4. Quantification of IL-17+ and IFNγ+ cells within CD4+CD161+ T-cells in gingival tissue. Gingival tissue cells were isolated from inflamed gingival tissue samples from patients with periodontitis (n=4). Samples were digested with collagenase. Cells were stimulated ex vivo with PMA and ionomycin for 3 hours prior to intracellular cytokine staining. Representative gating strategy to identify CD161+ vs. CD161- CD4+ T-cells and the percentages of IL-17+ and IFNγ+ cells within these populations.

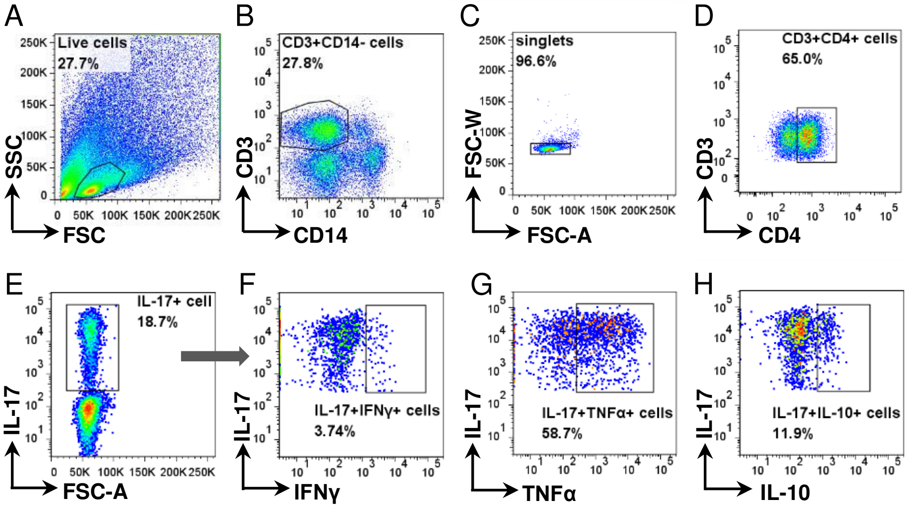

Supplementary Figure 5. Gating strategy for enumeration of cytokine co-expressing CD4+ T-cells in diseased gingival tissue. Gingival tissue cells were isolated from an inflamed gingival tissue sample from a patient with periodontitis. Samples were digested with collagenase. Cells were stimulated ex vivo with PMA and ionomycin for 3 hours prior to intracellular cytokine staining. The following gating strategy was employed: first, the live lymphocyte population was gated (A), then CD14-CD3+ cells were gated (B), then doublets were excluded based on FSC-W vs. FSC-A (C), and then CD3+CD4+ cells were gated (D). A gate was set on cells that stained positive for the cytokine IL-17 (E), and subsequently the percentages of IL-17+ CD4+ T-cells that co-expressed IFNγ, TNFα or IL-10 were determined (F-H).

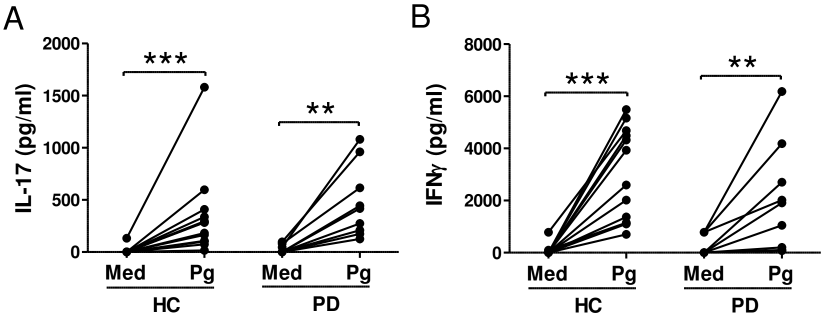

Supplementary Figure 6. Heat-killed Pg induces IL-17 and IFNγ production by CD4+ T-cell co-cultured with monocyte in the absence of anti-CD3 mAb. CD4+ T-cells (0.5x10^6 cells) and CD14+ monocytes were isolated from PBMC of periodontally healthy donors (n=13) or periodontitis patients (n=9), and co-cultured at a 1:1 ratio in the absence of soluble anti-CD3 mAb with or without heat-killed Pg (MOI=100) for 7 days. IL-17 (A) or IFNγ (B) production in culture supernatants was measured by ELISA. Each symbol represents an individual donor. Data were analysed by Wilcoxon matched-pairs signed rank test. **P<0.01, ***P<0.001.

Supplementary Table 1. Demographic and clinical characteristics of the study subjects

(A) Demographic characteristics of the gingival tissue sample donors[a]

	Healthy controls (n=6)	Periodontitis patients (n=12)
Age, mean years (range)	41.6 (26-66)	47.3 (37-66)
Female, no. (%)	1 (16)	3 (25)

(B) Demographic and clinical characteristics of the cohort study subjects[b]

	HC (n=13)	CP (n=15)	AP (n=15)
Age, mean years (range)	36.8 (20-63)	42.1 (36-58)	30.5 (19-36)
Female, no. (%)	7 (54)	8 (53)	7 (47)
Number of teeth, mean	28.7	28.1	28.8
PD, mean ± SEM mm	1.73 ± 0.24	3.53 ± 0.69	3.09 ± 0.79
BOP, mean ± SEM %	10.9 ± 7.0	32.1 ± 14.9	31.7 ± 29.3

a) Gingival tissue samples from patients with periodontitis (n=12) were obtained during periodontal surgery. Inclusion criteria for diseased tissues were presence of inflammation (bleeding on probing) and attachment loss of ≥ 6 mm. Healthy tissue samples (non inflamed) were obtained from periodontally healthy control subjects (n=6) undergoing non-periodontal disease related procedures such as crown lengthening or tooth extraction.

b) Peripheral blood samples and GCF samples were obtained from 15 patients with chronic periodontitis, 15 patients with aggressive periodontitis and 13 periodontally healthy control subjects. AP, aggressive periodontitis patients; BOP, bleeding on probing; CP, chronic periodontitis patients; HC, periodontally healthy controls; PD, probing depth.
